# Epigenetics and phenotypic variability: some interesting insights from birds

**DOI:** 10.1186/1297-9686-45-16

**Published:** 2013-06-11

**Authors:** Laure Frésard, Mireille Morisson, Jean-Michel Brun, Anne Collin, Bertrand Pain, Francis Minvielle, Frédérique Pitel

**Affiliations:** 1 INRA, UMR444, Laboratoire de Génétique Cellulaire, Castanet-Tolosan F-31326, France; 2 ENVT, UMR444, Laboratoire de Génétique Cellulaire, Toulouse F-31076, France; 3 INRA, UR631, Station d'Amélioration Génétique des Animaux, Castanet-Tolosan F-31326, France; 4 INRA, UR83, Recherche Avicoles, Nouzilly F-37380, France; 5 INSERM, U846, INRA, USC1361, Institut Cellule Souche et Cerveau, Bron F-69500, France; 6 INRA, UMR1313, Génétique animale et biologie intégrative, Jouy-en-Josas F-78350, France; 7 AgroParisTech UMR1313, Génétique animale et biologie intégrative, Jouy-en-Josas F-78350, France

## Abstract

Little is known about epigenetic mechanisms in birds with the exception of the phenomenon of dosage compensation of sex chromosomes, although such mechanisms could be involved in the phenotypic variability of birds, as in several livestock species. This paper reviews the literature on epigenetic mechanisms that could contribute significantly to trait variability in birds, and compares the results to the existing knowledge of epigenetic mechanisms in mammals. The main issues addressed in this paper are: (1) Does genomic imprinting exist in birds? (2) How does the embryonic environment influence the adult phenotype in avian species? (3) Does the embryonic environment have an impact on phenotypic variability across several successive generations? The potential for epigenetic studies to improve the performance of individual animals through the implementation of limited changes in breeding conditions or the addition of new parameters in selection models is still an open question.

## Review

Most economically relevant traits in animal production exhibit continuous phenotypic variations due to polygenic and environmental factors. Whereas many quantitative trait loci (QTL) have been identified for agronomic traits, in most cases, the underlying genes remain largely unknown. Genome-wide association studies have shown that, except for rare monogenic traits, the variability of complex traits is only partially explained by genetic variation [1]. Possible explanations include epistatic effects, structural variations, and insufficient detection power due to lack of individuals or markers [1,2]. Both epidemiological studies in humans and genetic studies in animals have revealed that, in addition to the DNA sequence, epigenetic marks may be transmitted across generations and influence the phenotype of offspring [3]. There are many discussions in the literature on what the term “epigenetics” refers to and this leads to numerous definitions [3-11]. While some definitions restrict epigenetics to modifications of the phenotype without changes of the DNA sequence that are transmitted to the next generations [4], other broader definitions include any form of information storage that maintains the DNA sequence intact, as described by Bird: "the structural adaptation of chromosomal regions so as to register, signal or perpetuate altered activity states" [7]. The former definitions link the term "epigenetic" to inheritance and the latter also refer to any phenomenon that leads to phenotypic plasticity. These two visions share a common feature i.e. the molecular mechanisms involved. The epigenetic machinery encompasses chromatin folding and its attachment to the nuclear matrix, packaging of DNA around nucleosomes, covalent modifications of histone tails, DNA methylation, and regulatory non coding RNA (such as miRNA, snoRNA, lncRNA). Epigenetic marks have been shown to actively contribute to the determination of patterns of gene silencing or active transcription, and to participate in the lineage and tissue-specific expression of genes [12-14]. Epigenetic marks are heritable from cell to cell through lineage development, and when acquired in early life, they can have an impact on the adult phenotype. They can also have an impact on the phenotypes of subsequent generations through multigenerational effects that occur either via epigenetic changes acquired during embryonic development, or through the inheritance of epigenetic marks via the gametes [3,15]. In this review, we retain the definition given by Feil and Fraga [14]: “Epigenetics is the study of mitotically and/or meiotically heritable changes in gene function that cannot be explained by changes in DNA sequence”.

Understanding the epigenetic regulation of gene expression due to environmental factors should provide important new insights into animal breeding, since the same genetic information may be used differently by individuals grown in different environments. However, epigenetic regulation of gene expression is not always environment-dependent as for parental imprinting in which parent-of-origin-specific expression of a subset of genes is regulated by epigenetic mechanisms. Examples of such loci have been documented in livestock species i.e. the locus responsible for the callipyge phenotype in sheep [16] and the locus that controls *IGF2* expression in pigs [17].

The first agricultural species to be fully sequenced was the chicken, however, to date, there are few studies on the relation between epigenetic processes and economically important phenotypes in birds. This review focuses on how epigenetic phenomena can have an impact on the adult phenotype of farmed birds.

The importance of sex-linked genes that account for some of the phenotypic variability has been shown in the chicken [18] and X inactivation that involves epigenetic mechanisms is well known in mammals [19,20]. In birds, only partial dosage compensation between the hetero- (ZW, female) and homogametic (ZZ, male) sexes has been described [21,22] and this was previously known as "lack of global dosage compensation" [23]. A region of hypermethylation (MHM for Male Hypermethylated Region, [24]) is associated with dosage-compensation of several genes in the male chicken [25-27], but not in zebra finch [21]. Many questions about the mechanisms of regional dosage-compensation still remain [26,28]. Since this topic has already been extensively reviewed, we refer the reader to the literature, including the references given above. By contrast, little is known about the mechanisms of genomic imprinting, if present, or developmental programming in birds although they may play a role in phenotypic variability as shown in mammalian farm animals. Similarly, epigenetic information that can be transmitted through several generations could have a significant impact on animal selection.

This review addresses the following questions: (1) Are there molecular mechanisms leading to genomic imprinting in birds? (2) While the influence of fetal environment on adult phenotypes is largely documented in mammals, what are the developmental and metabolic phenotypes due to specific environmental cues in birds? (3) Are there examples indicating that embryonic environment has multigenerational effects in birds?

### Genomic imprinting

**Figure 1:**
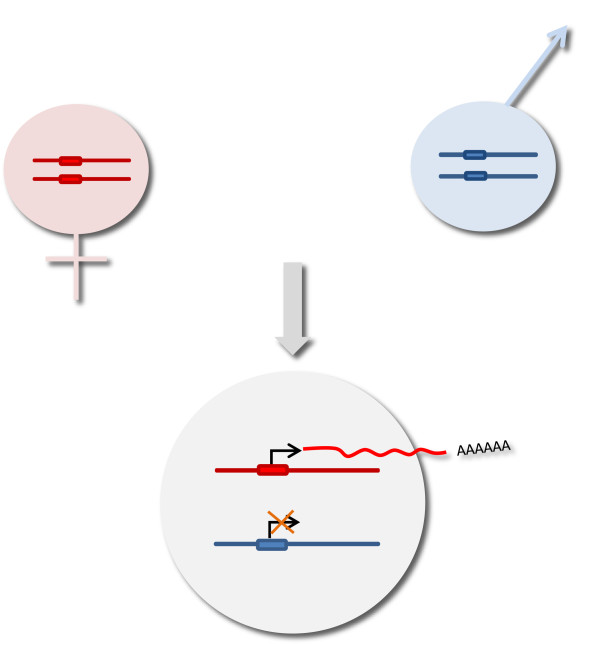
**Principles of genomic imprinting.** Each chromosome pair of an offspring consists of a maternal chromosome (in red) and a paternal chromosome (in blue). In this example, the offspring resulting from the cross expresses only its maternal allele (red), since the paternally inherited allele is inactive.

To discuss the state of knowledge regarding genomic imprinting in birds, the mechanisms known in mammals will be compared to the information available in avian species. To date, among vertebrates, genomic imprinting has been described only in eutherian mammals and marsupials. Parental imprinting (Figure 1), a process that leads to the differential expression of alleles depending on their parental origin (see [29] for a review), is stage- and tissue-specific [30,31]. The major theory explaining genomic imprinting is the parental conflict hypothesis [32,33], which states that the genes responsible for controlling the supply of maternal resources have a parentally biased expression, with the maternal genome tending to restrain resource allocations to preserve the mother and future progeny, while the paternal genome tends to facilitate this allocation to produce stronger offspring. Based on this theory, it can be assumed that genomic imprinting is restricted to organisms in which the maternal resources affect directly the embryonic genes, and thus its existence would be unlikely in oviparous animals [34].

In avian species, important reciprocal effects involving asymmetry in the contributions of the sire and dam to the offspring phenotype have been described for some traits. They explain 15 to 20% of the phenotypic variability in broiler body weight and egg viability in layers, and up to 47% in turkey egg production, and they have been extensively used to improve production in layers by designing optimized mating schemes [35]. These effects are mainly due to sex-linked genes, underlying the importance of chromosome Z in epigenetic effects, direct maternal effects ("larger females produce larger eggs") or mitochondrial DNA transmission [18]. However, this does not exclude the hypothesis that some of these effects may originate from parent-of-origin preferential allelic expression [36,37], and efforts to identify the genes involved in quantitative traits are increasingly taking epistatic and epigenetic effects into account. In the chicken and quail, many parent-of-origin QTL have been detected for traits linked with production [37-41], immune responses [42] and behavior [43]. However, such studies can detect spurious QTL due to linkage disequilibrium or bias generated by the experimental design [44,45]. A study reported by Rowe et al. [37] was specifically designed to avoid these biases i.e. it included a sufficient number of sires and dams to ensure segregation and a sufficient number of offspring to detect QTL with roughly equal allele frequencies in sires and dams for both QTL and molecular markers, and it fitted the common maternal environment in the linear model. Interestingly, this work confirmed the presence of a parent-of-origin QTL on chicken chromosome 1, in a region corresponding to orthologous imprinted regions in the human and mouse genomes. These results confirm the importance of studying genomic imprinting in birds.

Several studies have clearly demonstrated that some genes are paternally or maternally expressed during embryonic development in mammals [46-48]. Until recently, less than 200 imprinted genes were described (http://igc.otago.ac.nz/home.html) but a transcriptome sequencing approach reported in 2010 [49] uncovered parent-of-origin allelic effects for more than 1300 loci in the mouse. However, this large number is the subject of much debate [50,51], and to date, no consensus on the number of imprinted genes in mammals has been reached.

Some genes, known to be imprinted in mammals, have been examined in non eutherian vertebrates, in particular oviparous species [52], including birds, viviparous marsupials [53], and monotremes [54,55]. The *IGF2* gene, which has long been known to be paternally expressed in the mouse and man [56], has also been analyzed in the chicken. A preliminary report suggested that its expression is probably monoallelic [57], but later studies agreed that it is in fact biallelic [52,58-61]. In this case, analyses of different chicken tissues and at different growth stages led to divergent conclusions, emphasizing the importance of tissue sampling and time scaling in imprinting studies. The orthologs of other genes known to be imprinted in mammals, such as *ASCL2*/*MASH2* (a fully imprinted region in mammals), *M6PR*/*IGF2R*, *DLK1* and *UBE3A* were found to be biallelically expressed in the chicken [61-63]. Moreover, the *H19* imprinting center identified in mammals and controlling an imprinted cluster that includes *IGF2*, appears to be absent in the chicken [61].

However, such studies are limited to few genes (less than 5% of the genes known to be imprinted in the mouse) in different chicken embryonic tissues at different developmental stages. They are not sufficiently exhaustive to conclude that imprinting does not exist in birds, especially since different sets of genes might be imprinted in mammals versus birds.

Several studies have examined imprinted-related molecules and phenomena to better understand genomic imprinting in mammals. If applied to the chicken, this approach may also help test for the existence of genomic imprinting in birds.

One feature of imprinted regions in mammals is the asynchronous replication of parental alleles [64]. Interestingly, replication of the chicken orthologs of some imprinted genes is asynchronous [65], even when they are biallelically expressed. It is hypothesized that mammalian imprinted gene clusters originate from an ancestor common to all vertebrates and that they evolved from preimprinted to imprinted regions [66]. Since the orthologous mammalian imprinted genes are biallelically expressed in the chicken, it is impossible to strictly link the asynchronous replication to imprinting in birds.

Another feature of mammalian imprinting is its association with several molecular signatures. As recently reviewed, these signatures need to differentiate paternal and maternal inherited chromosomes in order to influence transcription, and to be transmitted through generations [67]. Changes in DNA methylation patterns represent an ideal mechanism to generate such signatures or epigenetic marks. DNA methylation is involved in the regulation of gene expression, and specific methylation patterns can be inherited across generations in mammals. The enzymes that control DNA methylation, such as DNA methyltransferases (DNMT), are crucial for embryonic survival in the mouse (recently reviewed in [68,69]). DNMT include proteins that act in the maintenance of DNA methylation, such as DNMT1, and proteins that are involved in *de novo* DNA methylation, either by directly interacting with DNA (DNMT3A and DNMT3B) or indirectly as supporting factors (DNMT3L) [70-74]. The respective roles of DNMT in genomic imprinting have been brought to light mainly through loss-of-function (knockdown or knockout) experiments [75,76]. Identification of methylation-related DNMT in chickens would stimulate the search for allele-specific expression in oviparous animals. *DNMT1*, *DNMT3A* and *DNMT3B* cDNA have been cloned in the chicken, and their encoded proteins have been shown to share 50-80% amino acid identities with the corresponding mouse orthologs [77,78]. However, *DNMT3L*, a gene that encodes a protein essential for the establishment of imprinted marks in the mouse [79] has not been detected in birds [77], which may explain why some genes imprinted in mammals are not imprinted in the chicken.

Another approach for investigating genomic imprinting is to explore the chicken genome for differentially methylated regions (DMR) that are involved in the differential methylation of maternal and paternal chromosomal DNA in mammalian imprinting. A genome-wide methylome map of chicken muscle and liver tissues was completed recently [80], and the authors did not identify any DMR associated with genes known to be imprinted in mammals. However, this search was performed for only a few genes in two tissues, and thus it is difficult to draw conclusions on the existence of imprinted genes across the chicken genome.

Although methylation patterns play a major role in the process of allele silencing, other mechanisms (e.g., histone modifications or non-coding RNA) are also known to be involved at several stages [81-84]. Among the numerous studies on this subject, two deserve particular attention. First, a study on mouse placenta has shown that genetic ablation of DNA methylation does not suppress imprinting of paternally repressed genes located in the distal region of mouse chromosome 7 [85] but that histone methylation seems sufficient to confer a silenced status to the paternal alleles of the relevant genes. The authors suggest the existence of an older imprinting mechanism that is limited to extra-embryonic tissues and that involves histone modification. In the second study, the authors examined the mouse *Gnas* cluster (located on mouse chromosome 2, containing a gene coding for stimulatory G-protein alpha subunit, giving rise to alternatively spliced isoforms that show maternal-, paternal- and biallelic expression as well as a non-coding antisense transcript [86]). They demonstrated that *Nespas*, a non-coding RNA, could silence *Nesp* by a mechanism independent of a DNA methylation mark [87]. Again, a DNA-methylation-independent role of chromatin marks in gene silencing was highlighted. These two studies show that imprinting mechanisms other than DNA methylation exist, and it is interesting to note that such mechanisms have not yet been investigated in birds.

Genome-wide approaches [49,88,89] and developments such as next-generation sequencing have recently opened up new perspectives for the investigation of imprinting mechanisms, including the possibility of identifying unknown mechanisms and gaining insight into new interactions or alternative processes. As suggested in a recent review [90], it is essential to explore other vertebrate lineages for epigenetic marks and allele-specific expression.

### Environmental epigenomics

The environment can influence developmental plasticity and thus phenotypes in a wide variety of animals, from insects to man [14]. Environmental epigenomics refers to the study of how environmental exposures (e.g., toxins, stress or maternal nutrition) during early development influence gene regulation through epigenetic mechanisms (e.g., DNA methylation or histone modifications) that, in turn, influence the adult phenotype [14,91-93]. As described below, the environment may have a much broader impact on the adult phenotype when the marks occur early during development.

#### Post-hatch environmental influences

Several studies on DNA and histone methylation levels in chicks subjected to heat stimulation demonstrated that epigenetic marks vary with the environmental conditions experienced during the post-hatch period [94-97]. They showed that the expression of BDNF (brain-derived neurotrophic factor), which is a key regulator of thermotolerance acquisition in the chick hypothalamus, differs between control birds and animals acclimated to heat early in their post-hatch life. Furthermore, alterations were observed in the methylation level of CpG sites in the promoter of the *BDNF* gene. It was also shown that modifications of histone H3 lysine 9 (H3K9) and methylation of histone H3 lysine residue 27 (H3K27) in the promoter of *BDNF* occur in the hypothalamus during thermotolerance acquisition on day 3 post-hatch.

Epigenetic modifications are involved in the immune mechanisms underlying chicken susceptibility to *Salmonella enteritidis*[98] or Marek disease [99,100] and include changes in the DNA methylation pattern of host defense genes. Indeed, the Marek disease virus (MDV) can induce changes in the expression levels of all three *DNMT* genes (*DNMT1*, *DNMT3A*, and *DNMT3B*). Various histone profiles and gene promoters were identified as being differentially modified and methylated in MDV-sensitive and -resistant chicken strains, indicating that epigenetic mechanisms may participate in the modulation of the resistance and/or susceptibility to specific poultry diseases [99,100].

Other environmental changes are known to affect the adult phenotype, but to date, no molecular evidence of epigenetic phenomena is available. For instance, phosphorus- or calcium-restricted diets during the early growing period trigger a compensatory adaptation of the chicken [101], possibly mediated by epigenetic mechanisms [102]. Although little is known about the underlying molecular mechanisms in birds, it seems that feed stress may alter gene transcription at least partly via epigenetic mechanisms. For example, Xu et al. [103] reported that 3-day-old chicks subjected to a 24-hour fasting underwent histone H3 methylation modifications in the preoptic anterior hypothalamus, which is the center of body temperature and food intake control.

#### Influence of the environment during development

In addition to post-hatch environmental factors, changes applied directly to the egg or the resources contained in the egg (e.g., nutrients, hormones, carotenoids, vitamins or RNA transcripts) can have an impact on newborn fitness and later on the adult phenotype [104,105]. Thus, these environmental effects on development are either directly applied to the embryo itself, or are transmitted by the mother.

In birds, direct abiotic environmental factors (e.g., temperature) can influence embryonic development and the adult phenotype (see [106]). It has been shown that exposure of embryos to different temperatures at the end of egg incubation, which is a critical developmental period, can be a way of adapting poultry embryos to later climatic conditions (see [106]). Epigenetic processes are good candidates for mediating these mechanisms [107-110]. Another example of abiotic stress influencing embryonic development is the exposure to green monochromatic LED light during embryogenesis that has a growth-promoting effect observed on adult turkeys and broiler chickens [111-114]. One explanation may be enhanced proliferation and differentiation of adult myoblasts and myofiber synchronization [113], but further work is needed to better characterize the underlying processes.

The impact of the mother’s environment on the F1 generation phenotype has been well documented in mammals. For example, it has been reported in humans, that the gestational diet affects offspring phenotypes (part of the "nutritional programming") (see [115]). A well-documented example comes from studies after the Dutch famine during World War II, which revealed that prenatal under-nutrition had an effect on later health [116] and that epigenetic mechanisms were involved [117]. The resulting chronic degenerative diseases associated with this famine include cardiovascular diseases, metabolic diseases, breast cancer and obesity. Another famous example of an adult phenotype induced by maternal nutrition in mammals is the viable yellow agouti (A^vy^) mouse model, in which the *Agouti* gene is genetically and epigenetically dysregulated by an upstream retrotransposon insertion. In this model, the diet of the mother influences coat color and other pleiotropic outcomes, such as diabetes, obesity and tumorigenesis in the offspring. Both methylation patterns and histone modifications are involved in the epigenetic variations of this mutation [118,119].

Maternal under-nutrition can also affect the phenotype of offspring in birds. For example, Rao et al. showed that 4-week-old chicks from mothers fed a low-protein diet had significantly heavier body weight and *Pectoralis major* muscle weight [120]. Another approach consists in experimentally increasing brood size, which induces developmental deficits (including nutrient deficits) in the early life of birds. For example, in zebra finches, Naguib et al. [121,122] imposed different degrees of developmental stress on nestlings by forming broods ranging in size from two to six nestlings, and then examined the offspring of the dams that had been differentially stressed as nestlings. As the brood size experienced by the dams increased, the weight of their offspring decreased. The effects on body mass and size were sex-specific. Female offspring grew larger than male offspring when their dam was raised in a small brood, but females from dams reared in large broods were smaller than their brothers. Furthermore, the reproductive success of the female progeny was negatively associated with the brood size in which the dam was raised [121,122]. These maternal effects could result from modifications in egg content of the females that were stressed during their early development, leading to sex-dependent impacts on the phenotype of F1 individuals. Epigenetic mechanisms might be involved in this process, and deserve to be examined in this context. Several studies have also proven the existence of a maternal influence on the immune system of F1 individuals in birds (see [123] for a review).

From a genetic point of view, it is interesting that the priming effect of these induced responses seems to depend on the maternal genetic background [124].

The most in-depth research on epigenetic effects in birds over a single generation focused on the effects of environmental challenges on behavioral traits, gene expression and DNA methylation in offspring [125-128]. In one of these studies [128], spatial learning was affected in individuals subjected to unpredictable light rhythms compared to animals exposed to predictable light rhythms. In the White Leghorn but not the Red Jungle Fowl, these effects were transmitted to the F1 generation reared under normal conditions, indicating a difference in the transmission of information to the next generation between these two chicken breeds. Exposure of commercial chicks to an unpredictable light schedule also triggered transmission of adaptive behavior to the next generation, with female offspring showing greater effects than males [127]. Molecular analyses showed that transcription differences acquired by the parents in response to environmental challenges are partially passed on to the F1 generation, and that the *BDNF* gene [128], immune genes [127], and stress-related genes [125] seem to be involved in these transmitted effects. This work also provides new insight into the role of DNA methylation in multigenerational epigenetic effects, by showing heritable differences of DNA methylation between different chicken breeds [126]. The influence of the genetic background is a particularly interesting feature and it has been reported that the impact of the parental environment on the offspring’s phenotype depends on the chicken line [128]. Observations in quail have also demonstrated a genetic component of maternal influence. Cross-fostering of chicks by mothers from two quail lines, divergently selected for tonic immobility (a fear-related behavioral trait), showed that the level of maternal influence on the offspring’s behavioral development depends on the chick’s genetic origin [129]. This maternal influence is at least partially carried by egg composition, as shown in a study of F1 quails from stressed females [130] and using an *ex*-*ovo* embryo transfer strategy between chicken layers and broilers [104].

**Figure 2:**
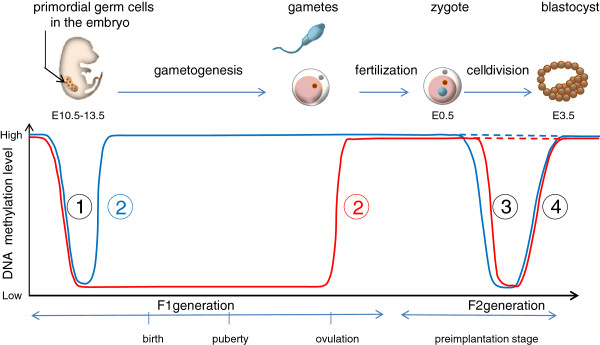
**The epigenetic genome**-**wide reprogramming cycle involves two phases of DNA erasure in the mouse (from [**[91]**,**[161]**,**[162]**]).** (1) A first wave of DNA demethylation takes place in the male (blue curve) or female (red curve) primordial germ cells (PGC) of the F1 individuals; this occurs throughout the genome, including the imprinted genes (embryonic day (E10.5-13.5). (2) Then, the genome of the gametes undergoes *de novo* methylation, with maternal methylation marks established at a later stage (ovulation) than paternal marks (E14). (3) A second wave of DNA demethylation takes place after fertilization in the F2 zygote (E0.5), with a more rapid demethylation in the paternal than the maternal genome. However, the paternal and maternal imprinted genes maintain their methylation pattern throughout this preimplantation reprogramming (dotted curves), allowing the inheritance of parent-specific monoallelic expression in somatic tissues of the F2 individual. (4) Finally, genome-wide remethylation occurs in both parental genomes at about the time of implantation (E3.5). Altogether the very early embryonic development corresponds to an epigenomic reprogramming step, during which the new epigenetic marks are more prone to being impacted by the environment. This explains why the environment experienced during early development has a greater impact on the adult phenotype than that experienced later in life [163]. Moreover, the timing of the two global DNA demethylation and remethylation waves differs between male and female genomes, possibly explaining why they may be differently impacted by a stress applied during these stages [91,164].

Collectively, the above-described examples yield two noteworthy conclusions. First, some of the early environmental effects on the offspring’s phenotype are sex-specific in both birds and mammals. Second, the environment experienced during early development seems to have a greater impact on the adult phenotype than that experienced later in life (Figure 2).

Taken together, these examples show that the environment influences gene expression in avian species, perhaps via epigenetic mechanisms. An interesting feature in the context of poultry production and selection is the possibility that these influences may be retained across several successive generations.

### Transgenerational memory of the ancestors’ environment

An example of transgenerational epigenetic transmission comes from the plant world. Johannes et al. showed that alterations in DNA methylation can be inherited for several generations in *Arabidopsis thaliana*[131]. Using epiRIL (epigenetic Recombinant Inbred Lines), these authors and others [132] examined the transmission of epigenetic marks for at least eight generations, and observed that some were conserved while others gradually returned to their original methylation state.

Similarly, interesting cases have been highlighted in animals. Erasure of methylation patterns during meiosis results in the establishment of new parent-specific imprints in oocytes and spermatocytes ( [15,133], see Figure 2). However, some loci can escape DNA methylation reprogramming, as for example, repeated elements such as retrotransposons [15,134]. Moreover, miRNA were shown to be involved in the transmission of epigenetic information via the gametes [15,135]. Thus, epigenetic information can be transmitted and have an impact on the next generations.

**Figure 3:**
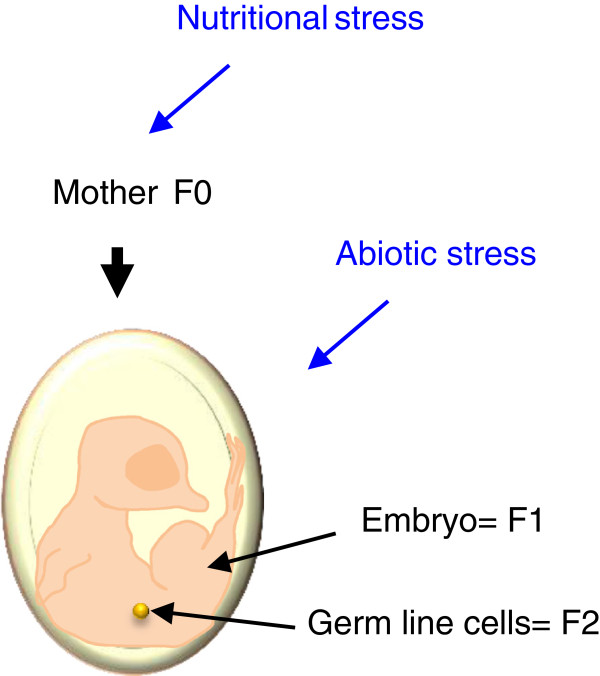
**The maternal environment may impact F1 and F2 individuals.** In birds, the maternal environment has an impact on individuals of the F1 generation through the egg content. However, it can also impact individuals of the F2 generation, since the developing offspring bears the primordial germ cells (PGC) that later differentiate into gamete precursor cells and finally lead to the individuals of the F2 generation.

Parental environment has an effect on the F1 generation and this is particularly clear in mammals, since the mother hosts the offspring’s development from the zygote stage to birth. Such effects will also occur in the F2 generation, since the developing F1 generation bears the primordial germ cells that will differentiate into gamete precursor cells and eventually form an F2 animal. In this way, the maternal environment can affect the next two generations (Figure 3), which means that the first generation for which an individual’s cells are not directly exposed to an environmental effect is the F3 generation if it was the female that was exposed and the F2 generation if it was the male. Thus, evidence for transgenerational epigenetic transmission, i.e. incomplete erasure of epigenetic marks between generations resulting in unusual patterns of inheritance from one generation to the next, is unquestionable only if the effect is detected in the F3 generation or beyond [136]. Investigating the male-path is an interesting approach to examine transgenerational epigenetic impacts.

Paternal environmental influences on the phenotype of the F1 generation (or even the F2 generation) have been shown in mammals (reviewed in [137]). For example, the female offspring of adult male rats fed a high-fat diet showed modified β-cell functions that were associated with an altered expression of more than 600 genes in the F1 generation, and hypomethylation of a cytosine proximal to the transcription start site of the *IL13RA2* gene [138]. Similarly, offspring from male rats fed a low-protein diet showed impaired lipid metabolism, notably associated with increased methylation at a putative enhancer of the *PPAR*α gene [139]. These results strongly suggest transmission of epigenetic information, but since the methylation patterns were not examined in the following generation, it is difficult to conclude to an unquestionable transgenerational epigenetic phenomenon, as defined above.

Transgenerational epigenetic transmission may be rare, but it has already been reported in different mammalian species. In man, Pembrey reported that the paternal grandfather’s food supply affected the mortality rate of grandsons but not of granddaughters, whereas the paternal grandmother’s food supply affected the mortality rate of granddaughters but not of grandsons [140,141]. Another study by Heijmans and collaborators showed that the risk of mortality in grandchildren, with respect to the grandparents’ food supply, was associated with modifications of DNA methylation in the differentially methylated region of the *IGF2* gene [117].

Recently, Zeybel et al. [142] described an adaptive mechanism involving epigenetic mechanisms in rats. After inducing liver injury in F0 and/or F1 males, they showed a reduction of liver fibrogenesis in F2 male offspring, illustrating an unquestionable transgenerational inheritance. The authors observed epigenetic modifications in a number of genes, with alterations observed in CpG methylation (*PPAR*γ, *PPAR*α and *TGF*-β*1*), histone H3 acetylation (*PPAR*γ and *TGF*-β*1*) and other chromatin modifications (*PPAR*γ). However, the mechanisms that transmit epigenetic modifications from the environment to the sperm and from the sperm to the offspring’s liver have not yet been deciphered [142]. In rats, an epigenetic inheritance induced by different environmental components was observed in the sperm of the F3 generation by detecting differentially methylated regions depending on the environmental exposure of the ancestors [143].

Some studies have even revealed transmission of epigenetic marks to at least the F4 generation. Recently, Wolstenholme et al. reported that exposure to bisphenol A during the gestation of female mice reduced the expression of the genes encoding two neuropeptides (oxytocin and vasopressin) in the brain of the F1 individuals. The expression of oxytocin was still reduced in the brain of the F4 males and females, whereas decreased vasopressin expression was maintained only in the F4 males. Moreover, impacts on social behavior were detected until the F4 generation [144]. Another report on the analysis of the phenotype and epigenetic marks of female rats subjected to a high-energy diet for four generations, demonstrated that transgenerational effects involving altered epigenetic marks at each generation were induced (at least partly) *de novo*[145]. Finally, the best-studied example of transgenerational epigenetic inheritance in vertebrates concerns the influence of vinclozolin on the health (fertility problems or organic diseases) of rat male offspring in the F1 to F4 generations. This occurs via DNA methylation and a putative induction of copy number variation to generate new imprinted-like sites that are transmitted to subsequent generations through the male germ line, thus creating transgenerational transmission of adult phenotypes [146-149]. Other studies have suggested putative intergenerational transmission of epigenetic marks through the gametes [15,135].

To our knowledge, no transgenerational transmission of epigenetic marks has been reported in birds, either prior to the exhaustive reviews by Jablonka and Raz [3] and Ho and Burggren [4], or since then.

## Conclusions

A phenotype results from the interplay between the genome and the epigenome, which itself depends on the environment the animal experiences during its development and adult life. Epigenetic variations during early life play a role in producing inter-individual differences in phenotypes. Consequently, analyses of inter-individual phenotypic diversity should consider both epigenetic and genetic variations [93]. In this review, we describe epigenetic phenomena in birds in comparison to the related studies in mammals. Much more work is needed to fully comprehend the importance of epigenetics in the phenotypic variability of birds, and hence to exploit it for genetic selection.

In the chicken, epigenetic modifications occur from the first egg stage, i.e. a stage at which the dam provides an environmental signature through the egg content [150]. These environmental influences may have agronomic value via their effect on the adult phenotype. Given the likelihood that climate will change in the more or less near future and demands for food supplies will increase, a better understanding of the epigenetic mechanisms governing the embryo’s response to environmental changes could open new ways to improve efficiency, animal welfare and food quality. For example, one interesting issue is the nutrient profile and restriction level of the diet of breeders, which is tailored to produce the largest possible number of fertile eggs and may thus not fill the requirements for future adult broiler performance [151].

Transgenerational inheritance associated with mechanisms other than DNA sequence variation (i.e., epigenetics, parental effects or "cultural inheritance") is thought to affect evolutionary dynamics [152]. This "non-classical" inheritance is known to play a role in phenotypic variability, especially in the response to environmental changes [153]. An important question in animal selection is the extent to which this non-genetic inheritance also affects the efficiency of genetic selection. Indeed, epigenetics may help to better explain environmental and non-Mendelian variability of complex traits [154]. Several authors have proposed quantitative models including epigenetic inheritance and environmental interactions [155-157], potentially paving the way for future inclusion of these mechanisms in genetic selection studies. The reversibility of epigenetic modifications (i.e., their potentially transient nature) could constitute a challenge in the modeling of inheritance [158]. Aside from putative epigenetic inheritance, Feinberg and coworkers proposed a model in which DNA mutations could, via epigenetic mechanisms, modify phenotypic variability without changing the mean phenotype [159]. This model should be considered by geneticists aiming at studying the adaptation of livestock to changing environments.

From a genetic point of view, the contribution of heritable epigenetic effects to important phenotypic variations is an exciting research area, not only for fundamental science, but also because of its possible breeding applications, as recently suggested by a primary poultry genetics organization [160].

## Competing interests

The authors declare that they have no competing interests.

## Author’s contributions

LF, MM and FP drafted and finalized the manuscript. JMB, BP, AC and FM participated in bibliographic analyses and writing of the paper. All authors read and approved the final manuscript.
